# Learning Reward Function with Matching Network for Mapless Navigation

**DOI:** 10.3390/s20133664

**Published:** 2020-06-30

**Authors:** Qichen Zhang, Meiqiang Zhu, Liang Zou, Ming Li, Yong Zhang

**Affiliations:** 1 Engineering Research Center of Intelligent Control for Underground Space, Ministry of Education, China University of Mining and Technology, Xuzhou 221116 China; zhangqichen@cumt.edu.cn (Q.Z.); liming@cumt.edu.cn (M.L.); 2 The School of Information and Control Engineering, China University of Mining and Technology, Xuzhou 221116, China; liangzou@cumt.edu.cn (L.Z.); yougzh401@cumt.edu.cn (Y.Z.)

**Keywords:** deep reinforcement learning, reward shaping, matching network, navigation

## Abstract

Deep reinforcement learning (DRL) has been successfully applied in mapless navigation. An important issue in DRL is to design a reward function for evaluating actions of agents. However, designing a robust and suitable reward function greatly depends on the designer’s experience and intuition. To address this concern, we consider employing reward shaping from trajectories on similar navigation tasks without human supervision, and propose a general reward function based on matching network (MN). The MN-based reward function is able to gain the experience by pre-training through trajectories on different navigation tasks and accelerate the training speed of DRL in new tasks. The proposed reward function keeps the optimal strategy of DRL unchanged. The simulation results on two static maps show that the DRL converge with less iterations via the learned reward function than the state-of-the-art mapless navigation methods. The proposed method performs well in dynamic maps with partially moving obstacles. Even when test maps are different from training maps, the proposed strategy is able to complete the navigation tasks without additional training.

## 1. Introduction

Autonomous navigation system enables the mobile robot to determine its position within the reference frame environment and move to the desired target position autonomously. The classical navigation solution is a combination of algorithms, including simultaneous localization and mapping (SLAM), path planning and motion control [1]. These methods rely on high-precision global maps, resulting in limitations in unknown map environments or dynamic environments. Recently, the research of mapless navigation, which implicitly performs localization and mapping, has attracted attention from both industry and academia [2]. Deep reinforcement learning (DRL) techniques, which map states to actions through continuous interaction with the environment, have achieved great success in many fields [3,4,5,6], such as video games, robot control and autonomous driving. A variety of DRL technologies have been introduced to mapless navigation tasks [7,8,9,10]. However, DRL algorithms are known to be data-inefficient [11], especially when the rewards are sparse. To accelerate the learning, dense rewards, which provide more information after each action are desired.

Reward shaping can be used to generate dense rewards [12], whose goal is that providing supplemental rewards to make a problem easier to learn. However, the design of manually programmed reward functions requires substantial domain expertise, especially in complex environments. Therefore, data-driven reward definition from trajectories or experiences has been studied [13,14,15,16,17]. Different from the hand-crafted reward function, inverse reinforcement learning (IRL) automatically learn reward function with trajectories from expert demonstrations [18,19]. However, IRL proved that expert demonstrations defeat the central goal of DRL: learning policies automatically by ‘trial and error’ [20]. Without expert demonstrations, data-driven methods for reward shaping directly use the sampled trajectories on a series of similar tasks. The approach of classifier-based reward shaping directly estimate the policy underlying sampled trajectories through pre-training a suitable classifier with supervised learning [20,21]. The probability of success in the current state obtained by the classifier will be used as the additional reward value. However, in order to ensure that the classifier can provide a suitable reward under any circumstances, a large number of samples are needed to train the classifier in a single task. To overcome such limitation, meta-learning has been introduced to design reward function or learn potential function [22,23,24]. However, the method assumes that the tasks share the same state space, and hence it might be ineffective when state space changes significantly. In addition, although the reward function based on meta-learning can learn a priori knowledge from different tasks, it still needs a certain amount of demonstrations under new tasks.

It was demonstrated that few-shot learning is able to learn from less samples than traditional deep learning methods [25,26,27,28,29]. In this paper, the matching network (MN) [30], a non-parametric approach to realize one-shot or few-shot learning, is proposed for designing a reward function in indoor navigation tasks. MN can implicitly extract the prior knowledge form trajectories on similar navigation tasks. Then the knowledge is transferred to new tasks in an additional reward manner.

To accelerate the learning speed of the navigation in a new environment, we propose a novel DRL framework based on MN. The main contributions of our framework lie in two folds.

We use the sequence composed of continuous robot states as the input of MN and the output of MN is the probability that the robot can complete the task. The benefit from the input is a sequence, MN can be applied to the navigation tasks where only partial information can be observed. The historical information contained in the path sequence enables the MN to make judgments about the current state of the robot.

Our method does not need human demonstrations or positive samples when training in a new environment, and the robot still can quickly learn the navigation strategy under the new map. The experiments demonstrate that our method has better adaptability to a dynamic environment and stable transfer ability. This mainly benefits from the experience gained by the reward function under other maps.

The rest of this paper is organized as follows. The related work of the proposed method is briefly introduced in the second section. The third section introduces our proposed method. The MN-based reward function structure and training process is introduced in Section 4. Section 5 introduces the structure of DRL model. The simulation experimental results of the model will be explained in Section 6. Section 7 presents a brief discussion and then concludes this paper.

## 2. Related Work

### 2.1. Navigation Algorithm

In the robot navigation problem, path planning is the foundation of robot navigation and control. Path planning generally consists of global path planning and local path planning [31]. Global path planning is to select a whole path when the map is known, which mainly including ant colony optimization (ACO) [32] and A-star algorithm [33]. Global path planning relies on known static maps and it may not work in the dynamic environment. Local path planning is to use the robot’s own sensors to obtain environmental information to complete obstacle avoidance and robot control. Commonly used methods include model predictive control (MPC) [34], artificial potential field [35] and the dynamic window approach (DWA) [36]. Due to the lack of global information, the result of local path planning may not be optimal.

Combine global path planning and local path planning, path planning is able to realize stable robot control based on known maps and robot models (kinematic models and dynamic models). It can find the optimal path in the known environment. However, the path planning algorithm needs to reset parameters when the environment or the robot changes, and it will consume a lot of calculation time to find a suitable path when the environment is more complicated.

### 2.2. Mapless Navigation

Mapless navigation refers to robots completing navigation tasks without artificially providing maps. Reactive approaches, which are able to control and execute a plan autonomously, were proposed for mapless navigation [37]. For instance, Roberge et al. [38] implemented particle swarm optimization (PSO) in real-time unmanned aerial vehicle (UAV) path planning. Zoumponos et al. [39] realized the path planning of the manipulator through fuzzy logic (FL). These reactive approaches are able to provide a path in unknown or dynamic environments. However, reactive approaches require a local map constructed using information obtained from the sensors. Therefore these methods cannot provide end-to-end control and they have several disadvantages such as longer computational time and complex design.

Recently, DRL is widely used in mapless navigation. It learns the mapping between agent states and actions to provide end-to-end control. A variety of DRL models such as Deep Q-Network (DQN) [40], deep deterministic policy gradient (DDPG) [41], asynchronous advantage actor-critic (A3C) [42] and proximal policy optimization (PPO) [43] have been applied and provided promising results. Long et al. [44] used laser data as input and proposed a PPO-based framework to avoid obstacles between multiple robots. Ma et al. [45] employed the RGB image as the visual input and presented a DRL-based mapless motion planner alleviating the need of interactions between the agent and environment. A few special techniques and model structures are also used in navigation tasks, including multiple subtasks to assist reinforcement learning [46], continuous motion control based on DDPG [47] and target-driven navigation [48]. Most of the above-mentioned methods focus on the improvement of reinforcement learning structure, and the reward value is mostly sparse. By designing a reasonable reward function, the effect of reinforcement learning can be further improved.

### 2.3. Meta-Reinforcement Learning

Meta-reinforcement learning (meta-RL) is able to rapidly learn new tasks. Duan et al. [49] used a series of interrelated RL tasks to train the recurrent neural network (RNN). Finn et al. [50] used model-agnostic meta-learning (MAML) to initialize parameters for DRL and DRL can quickly converge to the optimal strategy under new tasks. Unlike the previous work on training average models and model parameters, we focused on reward shaping. In recent years, several works have been investigated to improve learning efficiency for robots, including fast imitation learning [51,52] and meta-inverse reinforcement learning [53]. Both tasks are dedicated to quickly learning from a small number of demonstrations. Our goal is to achieve rapid and autonomous adaptation of the robot in the new environment without manually providing any new demonstrations.

### 2.4. Automatic Reward Shaping

Automatic reward shaping can reduce the dependence on the designers’ experience. Singh et al. [20] used the classifier combined with the active query mechanism, and only needs to provide few positive samples under a single task to achieve fast learning of DRL. This paper provides the classifier with a modest number of examples of successful outcomes, which can reduce the difficulty of sample collection. However, in the navigation problem, it is difficult to make the classifier judge by only providing the presentation whether the current state can complete the task. Xie et al. [21] used concept acquisition through meta-learning (CAML) [17] as the target code for DRL. After pre-training CAML under a variety of different tasks, the robot can obtain the ability of continuous learning with only providing a small number of demonstrations in a new task. However, humans must provide positive samples to CAML in new tasks and manually take the samples, which is time-consuming. Zou et al. [23] designed a reward function using meta-learning. It can quickly adapt to changes in a similar environment, but cannot perform well in the case of significant changes in the environment. Our method does not need artificial samples at the beginning of a new task. The robot collects samples through autonomous exploration and realizes the strategy of rapid learning to complete the task.

## 3. DRL with MNR

### 3.1. Problem Formulation

The aim of this paper was to design universal reward functions for DRL in navigation tasks. This problem is formulated as a Markov decision process (MDP). MDP is defined by a tuple ⟨S,A,T,R,γ⟩, where S is a set of state spaces, A is a set of actions, T is the transition probabilities, R is the reward function and γ is a discount factor. At each step, the agent receives the state $s_{t}=\langle o_{t},d_{t}\rangle$, where $o_{t}$ is laser scan data and $d_{t}$ is the Euclidean distance between the agent and the target point. We set the range of the $270^{\circ \;}$ laser scan as 0.1–10 meters. The agent action $a_{t}$ is sampled from a policy π, where $a_{t}=\left[v_{l}^{t},v_{w}^{t}\right]$, $v_{l}^{t}$ is the agent linear velocity and $v_{w}^{t}$ is angular velocity. In navigation tasks, the reward $r_{t}=R\left(s_{t},a_{t}\right)$ is usually calculated based on the Euclidean distance and the reward can be rewritten as $r_{t}=\alpha \left(d_{t-1}-d_{t}\right),\alpha >0$.

We considered an extension to the basic MDP framework, defined by ⟨$S,A,T,R_{ER}+R_{MN},\gamma$⟩, where the reward value of agent $r_{t}$ at each step is the sum of Euclidean distance reward $r_{ER}^{t}=R_{ER}\left(s_{t},a_{t}\right)$ and the additional matching network reward (MNR) $r_{MN}^{t}=R_{MN}\left(s_{1},s_{2},\cdots ,s_{t}\right)$. We denote this reward-augmented MDP as MDP(+MNR). In the following subsection, we will show that a policy that is optimal under $R_{ER}$ is also optimal under $R_{ER}+R_{MN}$.

### 3.2. Model Architecture

**Figure 1:**
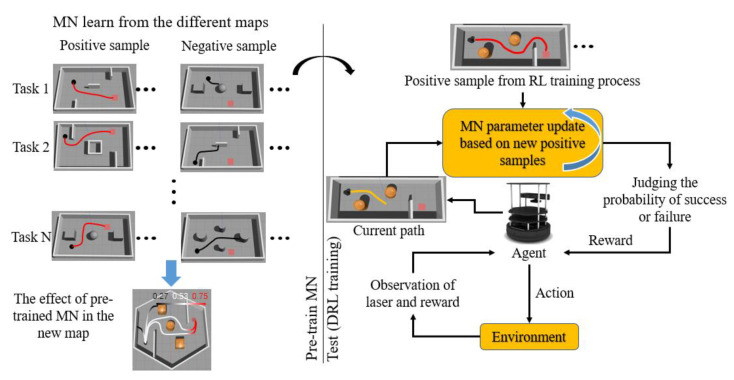
Our model flowchart.

In this research, we proposed a DRL method based on the MNR. The model flowchart is shown in Figure 1. Our reward function was designed based on MN. By pre-training the MN through different navigation tasks, MN is able to learn the rules of the navigation task. The pre-trained MN can generate a suitable reward in the new tasks. In the process of DRL training, the pre-trained MN evaluates the current state of the robot and generates rewards for guiding the DRL. After the robot completes a task, the newly obtained positive samples will be used for fine-tuning the MN, so that the reward value generated by the MN under the current map is more suitable.

## 4. Matching Network Based Reward

### 4.1. MN Network Structure

**Figure 2:**
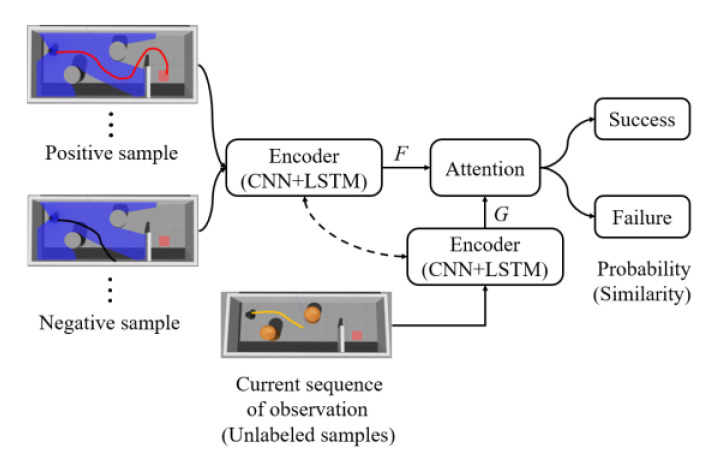
Matching network reward in mapless navigation.

Our reward function is based on MN, which combines attention and memory to realize fast learning. In the pre-training phase, MN learns the mapping between unlabeled samples $\hat{x}$ and labeled support sets of k examples $L={\left\{\left(x_{j},y_{j}\right)\right\}}_{j=1}^{k}$ thus MN can realize rapid adaptation under new tasks. The flow chart of MN in our method is shown in Figure 2. MN takes a set of state sequences as input and predicts the success probability of the current state. The state sequence $\left(s_{1},s_{2},\ldots ,s_{t}\right)$ is transformed to the input vector G through the encoder, which comprises of convolutional neural networks (CNN) and long short-term memory (LSTM). Meanwhile, MN selects the positive and negative samples from the data set as the input of the encoder, and gets the vector F. A similarity is given through computing the cosine similarity c between the vectors G and F. The attention mechanism is to use the softmax over cosine similarity and represented as:

(1) $$a\left(\hat{x},x_{j}\right)=e^{c\left(G,F_{j}\right)}/\sum_{j=1}^{k}e^{c\left(G,F_{j}\right)}$$

The sum of the softmax output values is 1. Each softmax output corresponds to a label and we added up all the softmax output that have the same label. Each support set contains two categories (positive and negative) and each category contains 20 samples. The output of MN is represented as:

(2) $$\left[j_{1},j_{2},\cdots ,j_{40}\right]\left[\begin{array}{c} \begin{array}{cc} 1 & 0\\ \vdots & \vdots \\ 1 & 0 \end{array}\\ \begin{array}{cc} 0 & 1\\ \vdots & \vdots \\ 0 & 1 \end{array} \end{array}\right]=\left[j_{1}+\cdots +j_{20},j_{21}+\cdots +j_{40}\right],\left(j_{1}+\cdots +j_{40}=1\right)$$

(3) $$Output_{MN}=\left(j_{1}+\cdots +j_{20}\right)$$

where $j_{i}$ is the softmax output. The labels of each sample in the support set are expressed in the form of one-hot. [1,0] represents the ‘success’ label and [0,1] represents the ‘failure’ label. We used $\left(j_{1}+\cdots +j_{20}\right)$ as the output of MN to represent the probability of success.

**Figure 3:**
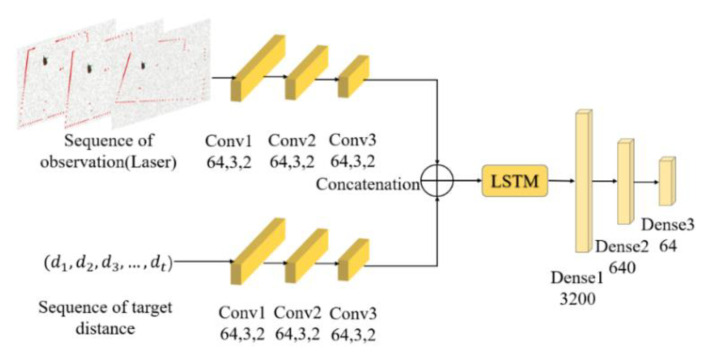
Encoder network structure. Every one-dimensional convolutional layer is represented by its channel size, kernel size and stride size. Other layers are represented by their types and dimensions. Each layer’s activation function is Rectified Linear Unit (ReLU).

Figure 3 shows the structure of the encoder. First, one-dimensional convolution was employed to respectively extract features of the laser scan data and the relative position relationship between the agent and the target position. Then we merged the compressed data and feed it to LSTM. The initial number of LSTM nodes was 128 and the step size was 25. 

### 4.2. MN Training Strategy

We pre-train MN with the data set collected from different maps. Each sample in the data set consists of a set of status sequence, which contains laser scan data and the Euclidean distance between the agent and the target position. In each training iteration, a fixed number of positive and negative samples were randomly selected from the data set as training set. Each training set included a support set and query set. After extracting features through the encoder, the similarity between the query set and the support set was calculated to obtain the label of the query sample (positive or negative). The errors between the similarity and label were used to update the MN parameters. The loss function of our model was the cross entropy and was represented as:

(4) $$loss=-\sum_{j=1}^{k}y_{j}*\ln p_{j}$$

where p is the output of MN.

The procedure pre-training MN is described in Algorithm 1.
**Algorithm 1. Pre-Training Matching Network (MN)**Input: Data set1: Initialize MN parameter *x*
2: For each iteration do3:  Sample training task from the data set, each task includes support set and query set4:  Support set and query set were coded to get *G* and *F*
5:  Calculate the similarity between *G* and *F*6:  Calculate the loss 7:  Update the parameters *x* through Adam optimizer

### 4.3. Sample Collection

**Figure 4:**
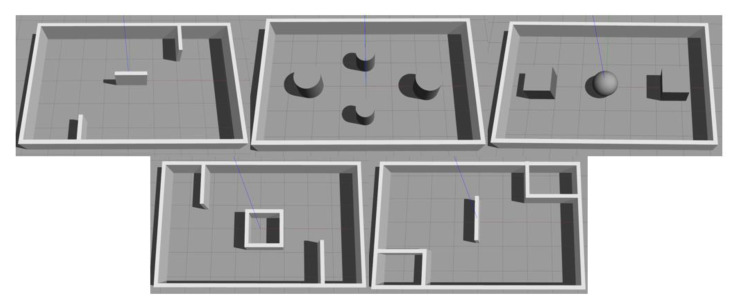
Data acquisition map (Pre-MAP).

Good-quality data is a prerequisite for training the MN well. Hence we collected a sequence of states on a variety of different maps to guarantee the variety of the data. The sample maps that we called Pre-MAPs are shown in Figure 4. The Pre-MAPs building in the Gazebo were 8 m long and 5 m wide. When we collected samples, starting and target positions in these maps were randomly generated. When the agent hits the obstacle (the smallest value in the laser scan data is less than 0.2) or runs over 200 steps, this process will be recorded as a negative sample. When the agent reaches the target position within 200 steps, this process will be recorded as a positive sample. Positive and negative samples are collected separately. When collecting positive samples, we used path planning methods to control robot to complete the navigation task. The path planning algorithm we used consists of the A-star algorithm and DWA. However, the path planning algorithm can only be applied when a map of the environment is known. When collecting negative samples, we set a random agent to interact with the environment. The robot’s initial positions and target point positions were randomly generated when collecting samples. In order to prevent the robot’s initial position from being too close to the target position, we set the Euclidean distance between the two points to be greater than a certain value. Under each Pre-MAP, we collected 60 positive samples and 60 negative samples, the total number of samples was 600. The data set collected under the Pre-MAPs is called the Pre-data set.

### 4.4. Pre-Training MN and Result Analysis

**Figure 5:**
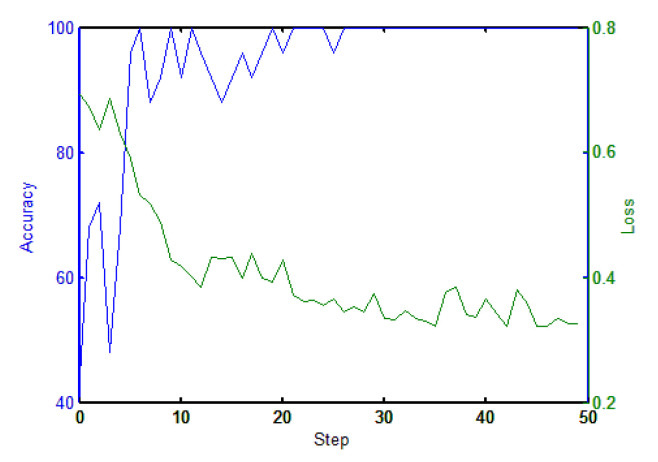
Matching network error and accuracy curve.

The MN used a 2-way 20-shot training method, where the samples in each training process contained two categories (positive and negative) and 20 samples were randomly selected for each category. The learning rate was set to $10^{-4}$ and the batch size was 20. The green and blue line in Figure 5 show the change in terms of loss and accuracy in the training process respectively.

**Figure 6:**
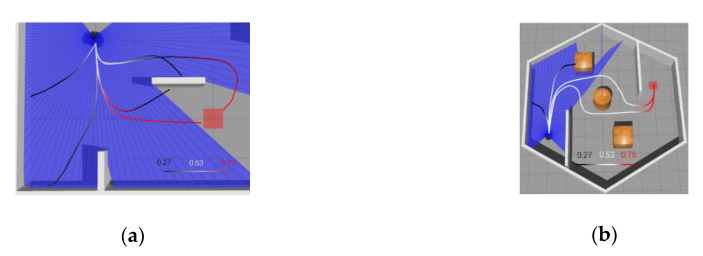
Matching network output changes on different paths: (**a**) matching network (MN) output under Pre-MAP and (**b**) MN output under new map without fine-tuning.

To determine whether the MN’s output can accurately evaluate the current status of the agent, we further tested output values on two maps under different paths for the robot. The results are shown in Figure 6. Figure 6a is one of the training maps and the Figure 6b is different from the training maps in terms of size and shape. Note that the shown output values are direct outputs from MN without any processing.

As can be seen from the Figure 6a, the MN yielded a result close to 0.5 when the robot just started running because the MN failed to determine whether the current state could succeed or fail. When the robot moved away from the target position, it could be seen that the given value decreased gradually. When the robot hit the obstacle, the MN gave an output value of only 0.27, which was considered as a failure. On the other hand, when the robot gradually approached the target position, the given value gradually increased, and before reaching the target position, the result given by MN was 0.75. It can be seen that MN had implicitly learned obstacle avoidance and can give a small output value when navigation task fails. In Figure 6b, MN is put into the new map without fine-tuning. The MN can also give a higher value when the robot is about to reach the target position. However, the majority of MN’s output is close to 0.5 when the robot is on the right path. It can be seen that after training the MN could still produce a suitable reward value without fine-tuning in a new environment, but it needs to be closer to obstacles or target points in order to have obvious changes. Therefore, during the initial training phase of DRL, MN can still provide some guidance.

## 5. Using MN-Based Rewards in PPO

### 5.1. PPO with MNR

We used PPO to make the robot generate a more robust strategy in the simulation environment, which was proposed by OpenAI in 2017 [43]. PPO, a new strategy gradient reinforcement learning algorithm, performs multiple gradient updates on each data sample, and hence it is more efficient and has better generalization ability than traditional strategy update methods. The parameters of PPO in the training process are updated as follows:

(5) $$\theta_{t+1}=\theta_{t}+\alpha \nabla_{\theta }J\left(\theta_{i}\right)$$

where J(θ) is the objective function of the PPO update, and J(θ) is represented as:

(6) $$J\left(\theta \right)=E\left[min\left(r_{i}\left(\theta \right)A_{i},\mathrm{clip}\left(r_{i}\left(\theta \right),1-\varepsilon ,1+\varepsilon \right)A_{i}\right)\right]$$

where $r\left(\theta \right)=\frac{\pi_{\theta }\left(a_{i}|s_{i}\right)}{\pi_{\theta_{old}}\left(a_{i}|s_{i}\right)}$ denotes the probability ratio between the two strategies before and after the update, $A_{i}$ denotes the estimated advantage. Clip function is the truncation function, which limits the value of r(θ) between 1−ε and 1+ε. PPO can effectively avoid a sudden change in strategy and ensure a stable training process by using clip function. In early stages of PPO training, the MN randomly selects samples from all trajectories in the Pre-data set since no positive samples are collected under the new maps. When positive samples under the new map is collected, MN will preferentially select samples from the new positive samples, and the rest are selected from the Pre-data set. With the increase of new positive samples, MN eventually only selects samples from the new positive samples. For the negative samples, MN randomly selects samples from the data set regardless of whether the samples are collected on new maps. The updated process of the PPO model is shown in Algorithm 2.
**Algorithm 2. Proximal Policy Optimization (PPO) with Pre-Trained MN Reward**Input: Pre—date set $L^{\prime }={\left\{\left(x_{i},y_{i}\right)\right\}}_{i=1}^{k}$, load the parameter χ of pre-trained MN1: Initialize PPO parameter θ and policy $\pi_{\theta }$
2: For each iteration I do3:  While the robot does not reach the target position do4:    Initializes the robot position5:    Get state $s_{t}$ (including laser scan state $o_{t}$ and relative position $d_{t}$)6:    Run $\pi_{\theta }$ generate action $a_{t}$
7:    Add $s_{t}$ to the queue ${\left(s_{1},s_{2},\ldots ,s_{t-1}\right)}_{I}$
8:    Input ${\left(s_{1},s_{2},\ldots ,s_{t-1}\right)}_{I}$ into MN9:    Get reward $r_{t}$ from MN and environment10:    Collect $\left\{s_{t}{,a}_{t}{,r}_{t}\right\}$
11:  Add ${\left(s_{1},s_{2},\ldots ,s_{t-1}\right)}_{I}$ and task result (Label) to the MN’s data set $L^{\prime }$
12:  If I is a multiple of 3 then13:    Update parameter χ of MN by $L^{\prime }$ with few epochs14:  Compute estimated advantage $A_{t}$
15:  Update θ with K epochs

**Figure 7:**
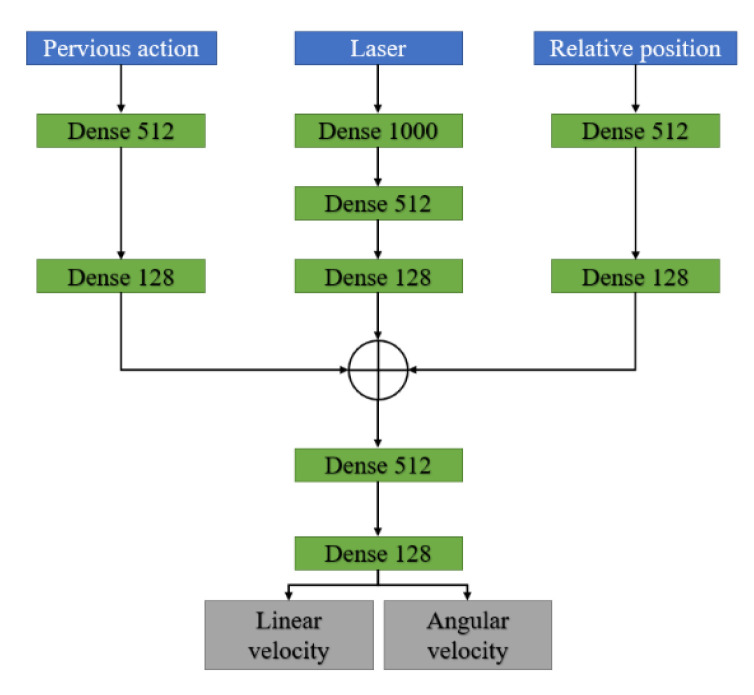
Proximal policy optimization (PPO) network structure.

The network structure of the PPO is shown in Figure 7. The PPO input $s_{t}$ and the action in the previous time-step $a_{t-1}$. The output of PPO is the linear and angular velocity of the robot. In the experiment we set the output of the linear velocity $v_{l}^{t}$ in [0,0.8], and angular velocity $v_{w}^{t}$ in [−0.5,0.5]. The network was composed of a fully connected layer and the activation function was ReLU. 

### 5.2. Reward Function Design and Policy Invariance

To map the $s_{t}$ to reward, we defined a potential function for the current timestep as $\phi \left(s_{t}\right),$ which is the output of the MN. According to potential-based reward shaping, the MN-based reward is defined as $r_{MN}^{t}=\gamma \phi \left(s_{t}\right)-\phi \left(s_{t-1}\right)\;\left(\gamma =0.96\right)$, which can satisfy optimal policy invariance [54,55]. The reward function of reinforcement learning is represented as:

(7) $$r\left(s_{t},a_{t}\right)=\{\begin{array}{c} r_{collision}=-50,if\;collision\\ r_{arrival}=100,if\;d_{t}<0.5\\ c_{r},if\;other \end{array}$$

where $d_{t}$ represents the Euclidean distance between robot and the target position. If the robot reaches the target position ($d_{t}<0.5$), the robot is given a positive reward $r_{arrival}$, which we set as 100 in the experiment. When the robot collided with the obstacle, we gave a negative reward $r_{collision}$, which was set as −50 in the experiment. To verify the effectiveness of our method, we compared several different methods. Among them, we compared CAML similar to MN. CAML, which is an extension of MAML, initialized parameters on different tasks to adapt on new tasks by using a small number of samples. Different from MAML, CAML only needs positive samples to adapt on new tasks. The similarities and differences between MN and CAML are shown in Table 1.

We compared several different reward functions as follows.

1. ER (Euclidean reward): in this process, we only used the Euclidean reward function, $c_{r}=r_{ER}^{t}=100\left(d_{t-1}-d_{t}\right)$, which is the benchmark for our tests.
2. MNR: in this step, we tested whether the matching network can provide effective guidance. The value generated by the MN was amplified as the reward function, $c_{r}=100r_{MN}^{t}$.
3. ER+CAML: in this step, we used the Euclidean and CAML as a reward function, $c_{r}=r_{ER}^{t}+100r_{CAML}^{t}$. Note that CAML was pre-trained by the samples described in Section 4.2. During DRL training, we artificially provided positive samples for fine-tuning of CAML.
4. ER+MNR (Ours): in this step, we combined the environment rewards function with MN, $c_{r}=r_{ER}^{t}+100r_{MN}^{t}$.

## 6. Navigation Experiment

To demonstrate the feasibility and adaptability of the proposed planning methods, various evaluation experiments were conducted. The experiment was divided into two parts: navigation under static map and navigation under dynamic map. Our experimental environment was based on Robot Operating System (ROS). In the experiment, the robot took 0.5 s to execute a step. We ran experiments on Intel Core i7-8750H CPU and Nvidia GTX1060 GPU with 16 GB of RAM.

### 6.1. Navigation Experiment with Static Obstacles

**Figure 8:**
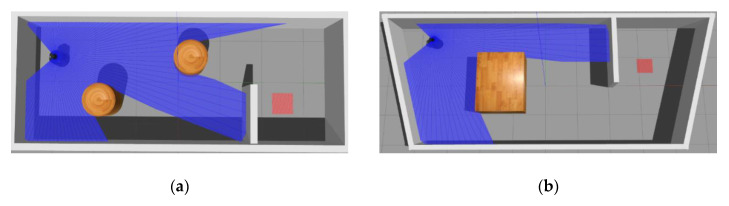
Two test maps (T-MAPs): (**a**) T-MAP 1 and (**b**) T-MAP 2.

As shown in Figure 8, we conducted robot navigation experiments under two maps (which we called T-MAPs) to evaluate the effectiveness of our approach. The first map is same with Pre-MAPs in size, which is 8 m long and 5 m wide, but the obstacles shape and location are different from Pre-MAPs. The second map is different from Pre-MAPs in size and shape. The map is an isosceles trapezoid, with a bottom length of 10 m, an upper bottom length of 8 m and a height of 4 m.

#### 6.1.1. MN Update

**Figure 9:**
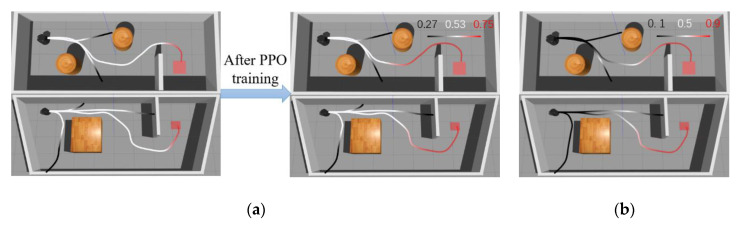
(**a**) Change of the output value produced by MN and (**b**) the output of CAML after fine-tuning.

During the PPO training, MN was also updating. After training, the average MN positive samples in T-MAP 1 increased by 181, and in T-MAP 2 increased by 230. Figure 9 shows the change of the MN’s output. Before PPO training, we found that pre-trained MN could give a reward value according to the current environment and the output value changes only when the robot was close to the target position or obstacle. As PPO continued to interact with the environment, MN could also get path samples under current map and update its parameters. When PPO training ends, we found the reward value produced by MN could give guidance to the robot. CAML is similar to the MN method, so in the Figure 9b we show the output value of CAML, which was fine-tuned with positive samples. However, we can see that the reward value given by CAML in Figure 9b was close to 0 when the robot had just started to run. This is because CAML was built on a CNN. At the beginning, the path collected by the robot was short, and the CNN tended to judge it as a negative sample. As a result, CAML could not provide effective guidance in the initial state of the robot.

#### 6.1.2. Results and Discussion

**Figure 10:**
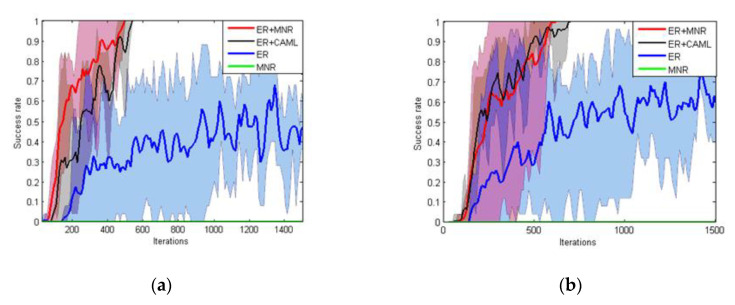
Success rate in training. (**a**) Success rate of the three methods in T-MAP 1 and (**b**) success rate of the three methods in T-MAP 2.

In order to have a well-rounded evaluation the impact of the reward function on DRL, we defined the following two metrics to evaluate the training results of DRL:

1. Success rate: the success rate means the success rate of navigation task of the last 25 iterations. Starting with the 30th iteration, we recorded the success rate every 10 iterations. In Figure 10, the *X*-axis represents the number of training iterations and the *Y*-axis represents the success rate. If the accuracy reached 100%, the robot could be considered to have completed the navigation task.
2. Training iterations: training iterations means the number of iterations that the robot trains when the success rate reaches the maximum in 1500 iterations of training.

We trained each method 5 times separately. The results under two different maps are shown in Figure 10. The solid line in the figure represents the average accuracy of five experiments. In our experiment, the robot stopped training when it reached a 100% success rate.

**Figure 11:**
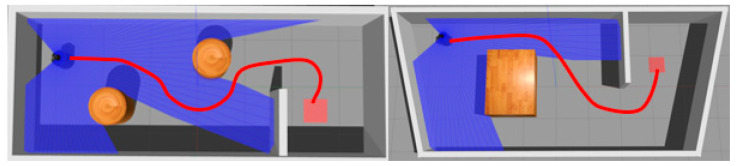
Model path.

From Table 2, it also can be seen that reward function based on ER+MNR could effectively improve the training speed of PPO. The main benefit of MNR was that it could accelerate the training speed in new tasks of RL without providing samples manually. As shown in Figure 9, the majority of pre-training MN’s output was close to 0.5. Hence, the reward still was ‘sparse’ and the success rate with MNR was always 0 until 1500 iterations. In the experiment we found that the robot was always circling around the initial position. The introduction of ER made up for the deficiency of MN at the beginning. Meanwhile, ER could only feedback the distance between the robot and the target position but could not encode the obstacle information. On the other hand, MN could guide the robot to avoid the obstacle and evolve to give a more dense reward with new positive samples. The performance of ER+CAML was slightly worse than that of ER+MNR. Figure 9 shows that the value given by CAML at the beginning was very low. If the robot hits an obstacle in fewer steps, then CAML cannot give the robot guidance under this path. As a result, ER+CAML training results were slightly worse. The path of the PPO model based on ER+MNR after convergence is shown in Figure 11. It can be seen from the Figure 11 that although there is still some gap between the path and the ideal optimal path (the minimum number of steps), it basically meets the requirements of navigation.

#### 6.1.3. Generalization Test on New Maps

**Figure 12:**
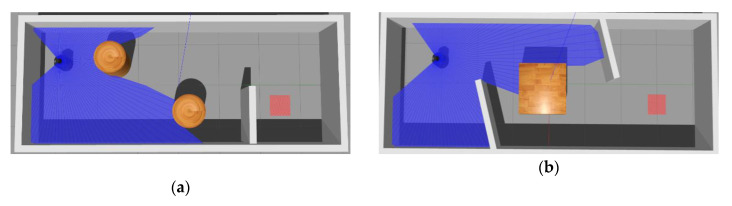
Changed four maps: (**a**) GT-MAP 1 and (**b**) GT-MAP 2.

To test the generalization capabilities of the trained policies with ER+MNR, we partially modified T-MAP 1 to become testing maps where the navigation paths was changed. Testing Map 1 (GT-MAP 1) was same as T-MAP 1 in size. GT-MAP 2 had a narrower accessible region, where the shape of the internal obstacles and navigation paths were completely different from T-MAP 1. These new maps are shown in Figure 12.

The robot directly deploys the ER+MNR-based policy and the ER+CAML-based policy trained on T-MAP 1 to navigate on new maps. We tested 100 times and recorded the success rate in each map. Networks parameters were not updated during testing. The results are shown in Table 3, from which we could conclude that proposed model performed as well as other methods in similar maps. 

**Figure 13:**
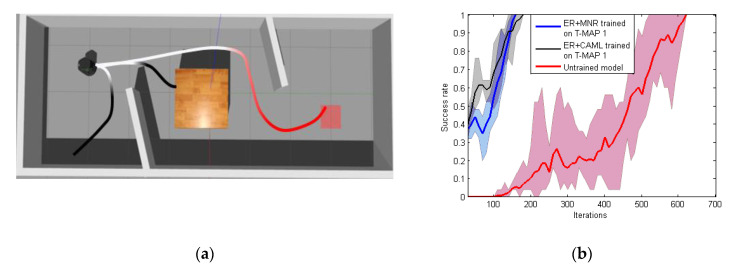
(**a**) Output value produced by MN in GT-MAP 2 and (**b**) training results.

However, in GT-MAP 2, which state space changes greatly, we found that the success rate greatly decreased. Whether the proposed model can quickly adapt to the state space change is also important. As shown in Figure 13a, the evolved MN from T-MAP 1 could directly give better output values in GT-MAP 2. We trained two ER+MNR-based policies on GT-MAP 2 respectively, one was based on the model trained on T-MAP 1, whose initial network parameters was the same as the trained correspondences in T-MAP 1. The other was the untrained model. The experimental results are shown in Figure 13b. With the help of prior knowledge, the trained model could achieve convergence only through average 134 iterations, which illustrates that our model could quickly adapt to navigation tasks under different maps. It can also be seen that the performance of our method was close to ER+CAML.

### 6.2. Navigation Experiment with Dynamic Obstacles

**Figure 14:**
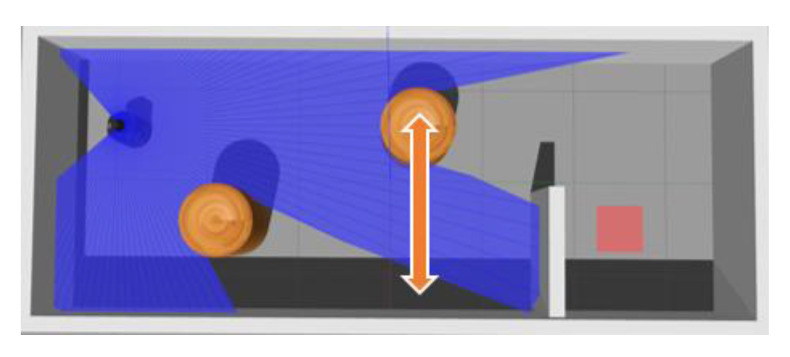
Map-dynamic.

There are usually dynamic obstacles in navigation tasks. Through MN is pre-training among the maps with static obstacles, we also tested the model in a dynamic environment, which is shown in Figure 14. The moving speed of the obstacle was 1.3 m/s.

For the experiments under dynamic maps, we tested the effects of the initial strategy training under dynamic maps and the effect of transferring to dynamic maps under static maps. The results are summarized in Table 4, from which we could conclude that ER+MNR learnt better than ER and the success rate of ER+MNR was 89%. At the same time, ER+CAML was able to achieve a 72% success rate under dynamic map training. Note that we provided CAML with positive samples of static maps to CAML at the beginning of ER+CAML training. As can be seen from Section 6.1.1, the initial reward value provided by CAML was close to 0, resulting in the output of CAML always being low until it encounters a closer obstacle. Therefore, CAML cannot let the robot know the information of nearby obstacles when the robot starts to run. The reward value given by MNR at the beginning is relatively neutral, and it will be reduced when approaching the obstacle. This process will make the robot understand the information of the obstacle, so the ER+MNR performs better on the dynamic map.

**Figure 15:**
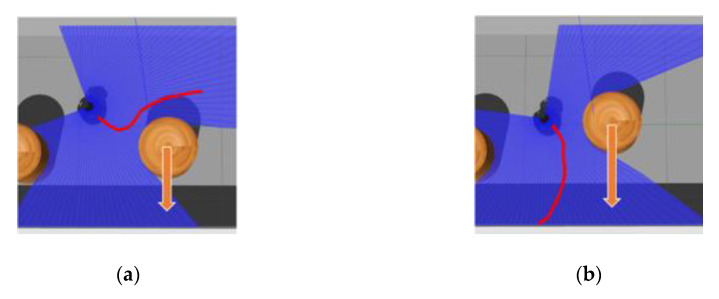
Strategy of ER+MNR when the robot encounters dynamic obstacles: (**a**) success and (**b**) failure

Figure 15 shows the strategy of ER+MNR when the robot encounters dynamic obstacles. In Figure 15a, the robot could turn quickly after approaching the obstacle, and continued to advance toward the target position. We also found that the effect of ER+CAML on dynamic maps was worse than ER+MNR. In the experiment we found that ER+CAML could not effectively avoid dynamic obstacles when it encountered the situation in Figure 15a. In Figure 15b, the robot’s moving direction was almost parallel to the obstacle moving direction. The robot only considered that the dynamic obstacle was static every time and tried to avoid collision. Unfortunately, the fact that the obstacles moved slightly faster than the robot led the task to fail. We concluded that MN lacked the ability of predicting the moving direction of dynamic obstacle. As supplementary material, the video of the experiments can be found in https://youtu.be/rkfmWaWJccw.

We also tested the policy, trained on static T-MAP 1, on dynamic map and the success rate achieve 79%. We could further verify that the model could learn the rules of navigation from a static map.

## 7. Conclusion and Future Work

In this paper, we proposed a MN-based reward shaping model. The model obtained the additional reward by comparing the current path of the robot with the provided samples. The obtained reward value encoded navigation skills. The experiments results show that the reward function model based on MNR+ER could effectively improve the training speed of DRL. 

Although our method had achieved some results in speeding reinforcement learning training, it still needs to be improved in the following areas:

1. On the whole, the output value of MN was closer to the discrete value, and the guidance effect on the robot was poor, so other reward functions need to be introduced.
2. The robot performed well in a static environment, but in a dynamic map, the robot lacked the ability of predicting the movement of obstacles, which led to a slightly poorer navigation effect of the robot in a dynamic environment.
3. Our proposed model is universal and we will also apply the model to other fields in future work.

## Figures and Tables

**Table 1:** Similarities and differences between MN and concept acquisition through meta-learning (CAML).

	MN	CAML
Pre-training goals	The goal is to give the network the ability to measure the similarity between two samples.	The goal is to make CAML learn the optimal initialization by updating on different tasks.
Need fine-tuning	MN can achieve classification tasks without fine-tuning, but the values generated after fine-tuning are more accurate.	Yes
Fine-tuning process	MN predicts new samples, and the error between the predicted value and the label is used to update the network parameters.	CAML updates parameters by one or a few steps of gradient descent with a few positive examples from the new task.

**Table 2:** Training effects on different maps.

	T-MAP 1		T-MAP 2	
	Average Maximum Success Rate	Average Training Iterations	Average Maximum Success Rate	Average Training Iterations
ER+MNR	100%	500	100%	620
ER+CAML (Provide positive samples manually)	100%	520	100%	680
ER	64%	1350	74%	1420
MNR	0	/	0	/

**Table 3:** Generalization test on new maps.

The Map	The Success Rate of ER+MNR	The Success Rate of ER+CAML
GT-MAP 1	85%	85%
GT-MAP 2	36%	42%

**Table 4:** Success rate under dynamic map.

	Average Maximum Success Rate	Average Training Iterations
ER+MNR	89%	1053
ER+CAML	72%	1120
ER	69%	1380
ER+MNR trained in T-Map1	79%	Untrained
ER+CAML trained in T-Map1	52%	Untrained

## Supplementary Materials

The following are available online at https://www.mdpi.com/1424-8220/20/13/3664/s1, Video: The strategy of ER+MNR when the robot encounters dynamic obstacle.
